# Duodenal lipoma: robotic-assisted approach

**DOI:** 10.1093/jscr/rjad220

**Published:** 2023-05-04

**Authors:** Ryan Alghanemi, Zülküf Tekin

**Affiliations:** Department of Surgery, KMG Kliniken Luckenwalde, Luckenwalde 14943, Germany; Department of Surgery, KMG Kliniken Luckenwalde, Luckenwalde 14943, Germany

**Keywords:** Lipoma, duodenum, Robotic surgery, Gastrointestinal tumors

## Abstract

Lipomas are one of the most common benign tumors of the gastrointestinal tract, typically found in the small and large intestine. While most cases are asymptomatic and discovered incidentally, large duodenal lipomas are rare and present a unique set of diagnostic and management challenges due to their complex anatomic relationships with other vital structures. Endoscopic removal of large lipomas carries a risk of bleeding and can be difficult to access. To address these issues, robotic-assisted surgical approaches have been proposed as a viable alternative to laparoscopy, as demonstrated in this case.

## INTRODUCTION

Large Duodenal Lipomas are uncommon and pose many diagnostic and management challenges due to their complex anatomic relationships with other vital structures. Symptoms usually arise when the Lipomas are larger than 4 cm and can include heartburn, feeling of fullness, bleeding, abdominal pain and even complete gastric outlet obstruction. Endoscopic removal of large Lipomas is not recommended due to the risk of bleeding and perforation [1, 2] . Therefore, surgical intervention is the preferred option for treating such cases. Robotic-assisted surgical intervention, as presented in this case, is a viable alternative to a laparoscopic approach [3] and could offers improved precision and accuracy, allowing for a more successful outcome.

## CASE REPORT

**Figure 1:**
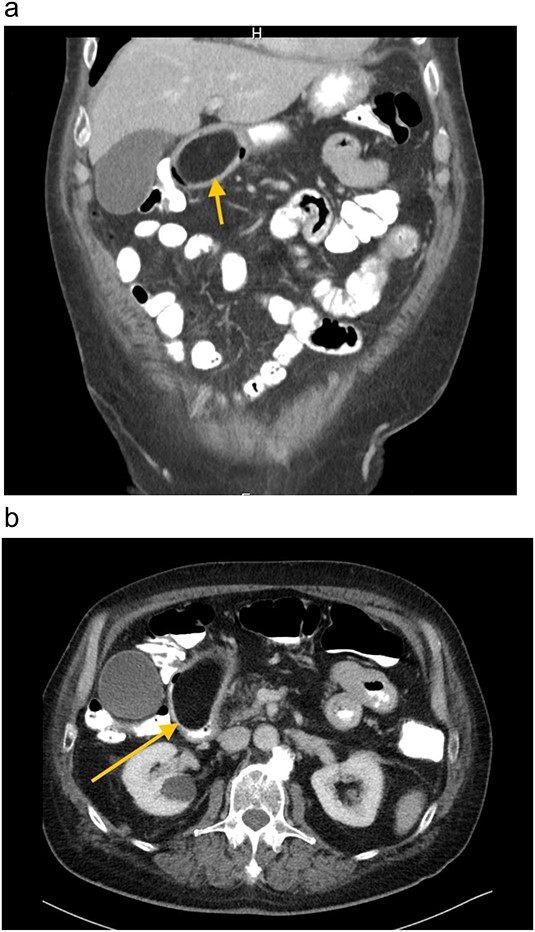
**(a)** and **(b)** CT-Abdomen, showing duodenal mass ⁓7.4 × 4.4 cm pressing the Pylorus.

**Figure 2:**
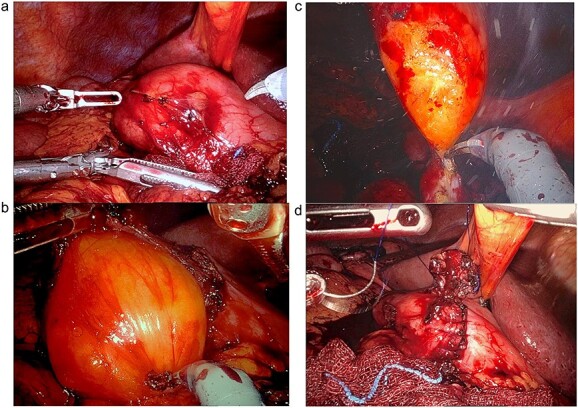
**(a)** After surgical preparation, the duodenum is exposed and prepared through Kocher maneuver for robotic-assisted duodenotomy. **(b)** The Lipoma is revealed after the duodenotomy is completed. **(c)** The Lipoma is then transduodenally resected. **(d)** The duodenotomy is then repaired in two layers, transversely.

A 67-year-old white German man with a known history of fully treated rectal cancer in 2011 presented with weight loss (13 kg in 3 months), night sweats, constipation and a feeling of fullness after small meals. Liquid diet was well tolerated. A computer tomography (CT) scan done 4 months prior to the presentation showed an incidentally discovered duodenal mass ⁓7.4 × 4.4 cm pressing the Pylorus without signs of inflammation or lymphadenopathy (Fig. 1). All related laboratory values were within the normal range. Subsequent endoscopic gastroscopy demonstrated Pylorus compression with a lipoma-like structure in the first part of the Duodenum. Due to the large size of the mass and the risk of bleeding, as well as the limited endoscopic access to the mass, robotic-assisted duodenotomy was performed as the treatment (Fig. 2). Histology revealed a benign lipomatous tissue, which was successfully removed via surgical excision. Postoperative follow-ups revealed a marked improvement in the patient's symptoms, along with a notable weight gain.

## DISCUSSION

Lipomas of the gastrointestinal tract are uncommon, slow-growing, fatty tumors that can occur anywhere along the gut. They are most commonly seen in individuals aged between 40 and 70 [2]. The tumor itself is composed of well-differentiated adipose tissue surrounded by a fibrous capsule, and has a yellow, lobulated cut surface with a gross appearance of subcutaneous fat. In total, ⁓90 of lipomas are located in the submucosa, while the remaining 10% are subserosal or intramucosal [4]. Due to its position immediately superficial to the muscularis propria, underlying muscular contractions can draw the tumor into the bowel lumen, forming an intraluminal polyp on a pseudopedicle. Large duodenal Lipomas present a great challenge to remove due to their complex anatomic relationships with other vital structures. In such cases, robotic-assisted surgery is a useful approach that should be considered and utilized [3].

## CONCLUSIONS

Symptomatic large duodenal lipomas are rare and present many diagnostic and management challenges. Robotic surgery has emerged as an effective alternative to laparoscopy and could offer superior outcomes in the hands of experienced surgeons. Therefore, robotic surgery should be considered as a viable option for the management of symptomatic large duodenal lipomas.
